# Enhanced mitochondrial respiratory metabolism in UV-damaged HDF cells based on metabolomics analysis of fermented *Galla rhois* gallnut extract

**DOI:** 10.3389/fphar.2026.1922913

**Published:** 2026-10-27

**Authors:** Jiaying Wu, Ning Wang, Denghui Chen, Yi Zhang, Jingxia Sun, Xiao Zhang, Guanlin Li, Xiang Li, Hu Huang

**Affiliations:** 1 Proya International Academy of Sciences, Proya Cosmetics Co. Ltd, Hangzhou, China; 2 Jinhua Institute of Zhejiang University, Jinhua, China

**Keywords:** fermentation, *Galla rhois*, metabolomics analysis, mitochondria, UVB

## Abstract

UV-induced photoaging disrupts mitochondrial homeostasis in skin cells, which in turn exacerbates skin aging. In this study, we developed a novel fermented *Galla rhois* gallnut extract (FGRE) using *Lactiplantibacillus plantarum* with mitochondrial respiratory homeostasis amelioration. Biochemical assays showed that FGRE exhibited stronger antioxidant and anti-elastase activities than the unfermented extract. Using Seahorse XF Cell Mito Stress Test, 1% FGRE significantly enhanced mitochondrial respiratory function in UVB-irradiated human dermal fibroblast (HDF) cells compared to that with the unfermented extract. In addition, 1% FGRE increased the ATP content and NAD^+^/NADH ratio and regulated the transcription level of fusion and fission genes in UVA-induced HDFs. Furthermore, the non-targeted metabolomics analysis revealed that differential metabolites were predominantly enriched in amino acid metabolic pathways, and 24 differential metabolites were putatively identified. Among these, the differential metabolites leucinic acid, urolithin A, and urolithin M5 were quantitatively determined. Notably, 1 μg/mL leucinic acid (equivalent to the concentration in 1% FGRE) ameliorated mitochondrial respiratory dysfunction and promoted the ATP production in UV-induced HDFs. Therefore, leucinic acid was considered as one of the potentially bioactive metabolites of FGRE.

## 1 Introduction

Skin aging is a complex biological process, which is intuitively presented as increased wrinkles, decreased elasticity, laxity, enlarged pores, and pigmentation of skin (Gu et al., 2020). In addition to endogenous aging, external environmental factors, especially ultraviolet (UV) radiation, accelerate the aging process (Rittié and Fisher, 2015). In the recent years, mitochondrial homeostasis has been considered crucial for skin barrier and skin health (Martic et al., 2023). Actually, emerging evidence has increasingly positioned mitochondrial dysfunction and oxidative stress as central drivers in UV-induced skin photoaging (Naidoo et al., 2018). Photoaging causes mitochondrial energy metabolic dysfunction, structural and morphological abnormalities, dynamics dysregulation, accumulation of mitochondrial DNA deletions, and the increase of reactive oxygen species (Sreedhar et al., 2020). These lead to a vicious cycle of increasing damage in skin cells, thus aggravating skin aging (Stout and Birch-Machin, 2019). Therefore, improving cellular mitochondrial function is considered an effective method to alleviate skin aging. Current conventional strategies to mitigate skin UV-induced damage mainly include topical sunscreens, exogenous antioxidants, and pharmacological agents. Topical sunscreens comprise physical (e.g., zinc oxide and titanium dioxide) and chemical (e.g., oxybenzone, avobenzone, and octinoxate) UV filters (Guan et al., 2021). Exogenous antioxidants, including vitamin C, vitamin E, ferulic acid, and coenzyme Q10, are widely used to scavenge UV-generated reactive oxygen species (ROS) and augment endogenous antioxidant defenses (Pandel et al., 2013). Retinoids, such as tretinoin and tazarotene, are prescription drugs approved by the FDA for the treatment of photoaging (Commander et al., 2016). Although these strategies are widely used to prevent or treat UV-induced skin damage, they are associated with several limitations, including incomplete photoprotection, formulation instability, and adverse effects. Consequently, natural product-based preventive or therapeutic approaches have drawn considerable attention.

*Galla rhois* gallnut extract (GRE) is a cosmetic raw material listed in the Inventory of Used Cosmetic Ingredients under China’s National Medical Products Administration. It is derived from a traditional Chinese medicine (also named Galla chinensis), which is the gall induced by the Chinese aphid (*Schlechtendalia chinensis*) on the leaves of *Rhus chinensis* Mill.*, Rhus potaninii* Maxim*.*, or *Rhus punjabensis* Stew. var. *sinica* (Diels) Rehd. et Wils. (Djakpo and Yao, 2010). The Chinese Pharmacopoeia records its function as astringing exudations and healing sores, indicated for skin maceration and exudation and carbuncles and sores (Chinese Pharmacopoeia Commission, 2022). Some studies reported that *G. rhois* had anti-inflammatory, hemostatic, and analgesic properties that could be used to promote clotting following traumatic injuries and to treat burns and skin infections (Lee et al., 2014). Ethyl digallates isolated from *G. rhois* suppressed LPS-stimulated NO production in RAW 264.7 macrophages *via* HO-1 induction (Park et al., 2011). A study showed that *G. rhois* extract could inhibit the elastase and tyrosinase activities *in vitro*, and it reduced the expression of MMP-1 in UVA-induced fibroblasts (Park and Park, 2010). Gallotannin-enriched *G. rhois* extract inhibited ROS production in H_2_O_2_-induced HepG2 cells (Go et al., 2017). In addition, Fang’s research showed *G. rhois* extracts obtained using five different extraction solvents all exhibited favorable *in vitro* antioxidant and antibacterial activities (Tian et al., 2009). These studies provided supporting evidence for the traditional use of GRE in treating skin disorders, along with their potential in ameliorating skin photoaging.

Fermentation is a key technology for food and medicinal plant processing for enhancing the nutritional and nutraceutical value. In traditional Chinese medicine, fermentation has long been used to process medicinal materials and improve their therapeutic properties. Baiyaojian is a traditional fermented preparation of *G. rhois* documented in classical Chinese materia medica. It is produced by fermenting *G. rhois* powder with tea and jiuqu (a traditional starter culture), during which microbial enzymes convert high-molecular-weight tannins into gallic acid and related smaller bioactive compounds. In modern practice, *Lactiplantibacillus (L.) plantarum* (formerly *Lactobacillus plantarum*, reclassified in 2020 b y Zheng et al.) is the most widely used starter for plant fermentation and is uniquely equipped with several enzymes, including hydrolases, esterases, decarboxylases, and reductases (Zheng et al., 2020; Muñoz et al., 2024). *L. plantarum* has an extensive history of safe use, holds the GRAS (generally recognized as safe) status from the U.S. FDA (Food and Drug Administration), as evidenced by multiple GRAS notices including GRN 685 (Probi AB), GRN 722 (DuPont), and GRN 1113 (Chr. Hansen), and is included in the EFSA QPS (European Food Safety Authority, Qualified Presumption of Safety) list. *L. plantarum-*fermented *Rhodiola rosea* activated the Nrf2/Keap1 signaling pathway and protected fibroblasts from oxidative stress caused by UVA, and the effect was significantly better than that of unfermented *R. rosea* (Fu et al., 2022). In addition, lactic acid bacteria’s metabolites or their extracts also have beneficial effects on the skin. For example, extracellular vesicles produced by *L. plantarum* reduced wrinkle formation, improved skin elasticity, increased skin moisture content, improved skin density, and inhibited skin pigmentation caused by aging in clinical trials (Jo et al., 2022). Therefore, applying *L. plantarum* fermentation to *G. rhois* represents a controlled, modernized implementation of the traditional Baiyaojian biotransformation principle, providing a reproducible strategy to enhance the bioactivity.

In this study, GRE was fermented with *L. plantarum* to obtain fermented GRE (FGRE). The FGRE was evaluated for its modulatory effects on mitochondrial function in UV-irradiated human dermal fibroblast (HDF) cells. The non-targeted and targeted metabolomics were used to analyze the metabolites of FGRE and combined with cell experiments to determine the potentially active metabolites. The results indicated that FGRE could improve the mitochondrial function, which provided a novel strategy for ameliorating UV-induced skin damage.

## 2 Methods

### 2.1 Chemicals and reagents

GRE (Batch No. Fh240527) was purchased from Focusherb Co. Ltd. (Shanxi, China), and its source was identified as the gall of *R. chinensis* Mill. *L. plantarum* PIAS240228 *was* isolated from fermented food by our laboratory and deposited at the China Center for Type Culture Collection (CCTCC, Wuhan, China) under accession number CCTCC NO: M 2024647. Fermented nutrient salt NS01 (containing yeast extract, phosphate, and trace elements, among others) was obtained from AngelYeast Co., Ltd. Leucine acid and leucinic acid were purchased from Shanghai Macklin Biochemical Co., Ltd (Shanghai, China). Urolithin C (UroC, 95% purity) and urolithin A (UroA, 98% purity) were obtained from Sigma (Shanghai, China). Urolithin M5 (UroM5, 96% purity) and urolithin M6 (UroM6, 92% purity) were purchased from NatureStandard Shanghai Standard Technology Co., Ltd. HDF cells purchased from Guangdong BioCell Biotech Co., Ltd. (Guangdong, China). All other chemicals of analytical grade were obtained from Sigma (Shanghai, China), unless otherwise noted.

### 2.2 Fermentation and preparation of FGRE

The strain *L. plantarum* PIAS240228 was inoculated into MRS medium for 2–3 times activation. The fermentation process was carried out with minor modifications based on the method described by Frank et al. (2006) (Frank et al., 2006). Briefly, *L. plantarum* was inoculated in GRE medium with 2% (v/v) inoculation (10^7^ CFU/mL) and fermented for 36 h–48 h. pH was controlled to 5.5 ± 0.2 using 10 mol/L NaOH. The culture condition was 37 °C and aerobic. The GRE medium contained 100 mg/L GRE, 10 g/L glucose, and 10 g/L fermented nutrient salt NS01 dissolved in 0.03 M potassium phosphate buffer (PBS, pH = 7.2). The fermentation broth (viable count ≥ 10^9^ CFU/mL) was homogenized and broken with 1,000 Bar for 20 min by high-pressure homogenization. Then, the final FGRE sample was obtained by centrifuging at 11,000×g for 10 min and concentrating 3–4 times. The GRE sample for follow-up experiments was also obtained by centrifuging at 11,000×g for 10 min and concentrating 3–4 times from the GRE medium. The FGRE and GRE samples were normalized to the same Brix level (7.5%) during the preparation process, and the dry-matter was determined by heating to 105 °C. In addition, GRE and FGRE samples were further explored for the effect and metabolite composition. The total phenolic content in FGRE and GRE was determined by the Folin–Ciocalteu method. The pH and soluble solids content (Brix) were measured in FGRE, aiming to ensure inter-batch reproducibility and stability.

### 2.3 Biochemical experiments

The antioxidant activity of FGRE was determined by 2,2-Diphenyl-1-picrylhydrazyl (DPPH) radical scavenging assay according to the method described by Lee et al. (2015) (Lee et al., 2015). Briefly, 100% (v/v) GRE and FGRE were mixed with an equal amount of DPPH solution and left to stand for 30 min at room temperature in the dark. Then, the absorbance of the resulting solution was determined at 517 nm. Water was taken as the control, while 12.5 μg/mL ascorbic acid (Vc) was taken as the positive control. The scavenging ability was defined as follows:

$$\text{Scavenging activity }\left(\%\right)=\frac{A_{\text{control}}-A_{\text{sample}}}{A_{\text{control}}}\times 100\%$$

The anti-elastase activity was measured according to Shirzad’s research with slight modification (Shirzad et al., 2018). A measure of 420 μL 0.05 M Tris–HCl (pH = 8), 180 μL of samples [100% (v/v) GRE and FGRE], and 150 mL elastase enzyme (600 mU/mL) were mixed together and incubated in room temperature (25 °C) for 15 mins. A measure of 150 μL N-succinyl-Ala-Ala-Ala-Val-*p*-nitroanilide substrate (1.015 mM/L) was added to each tube. After 20 min incubation in room temperature (25 °C), absorbance was read at 410 nm. Epigallocatechin gallate (EGCG, 500 μg/mL) was used as the positive control. The percentage inhibition was calculated as follows:

$$\text{Inhibition }\left(\%\right)=\frac{A_{\text{control}}-A_{\text{sample}}}{A_{\text{control}}}\times 100\%.$$

### 2.4 Mitochondrial respiratory measurement in UVB-induced HDFs

HDF cells from three independent donor lots (lot numbers: Fb23082101, Fb19052002, and Fb25081901) were used to explore the effect of FGRE on mitochondrion. To minimize technical variability, fibroblasts from all three donor lots were cultured and passaged in parallel, and all samples were tested concurrently in one independent experimental day. All experiments were performed using cells at passage 10 or less. The cytotoxicity of different samples was first evaluated using the CCK-8 assay. Subsequently, the experimental groups and treatment were performed as follows. On the first day, all incubated HDF cells were cultured in DMEM medium supplemented with 10% fetal bovine serum at 5% CO_2_ and 37 °C for 24 h. Then, the cells in the control group (NC) and UVB group were still cultured in DMEM medium supplemented with 10% fetal bovine serum for 24 h. The GRE and FGRE groups were cultured in 1% GRE and 1% FGRE for 24 h, respectively. On the third day, the cells in UVB, GRE, and FGRE groups were washed 2–3 times with PBS and exposed to UVB (0.30 mW/cm^2^) in PBS for 10 min (cumulative energy 180 mJ/cm^2^), while the NC group was not exposed to UVB. UVB (NB-UVB) was generated by a Narrow-Band UVB lamp (Philips, TL20W/01 lamps) with a peak at 311 nm. The emitted radiation was checked under the microplate using a radiometer (Solar Light PMA2100, USA). The dose of UVB exposure was selected based on the reference and preliminary experimental results (data not shown) (Zhou et al., 2025). After irradiation, PBS was replaced with DMEM medium, and the cells were cultured for an additional 24 h before testing. The oxygen consumption rate (OCR) in HDF cells was determined by a Seahorse Extracellular Flux (XF) analyzer (Seahorse Bioscience, USA), and the basal respiration, ATP production, maximal respiration, and spare respiratory capacity were analyzed according to Wang et al. (2015). Further, leucinic acid, UroA and UroM5 were also explored the effect on mitochondrial respiratory by the methods as mentioned above. The protein content was measured by commercial BCA kits (Thermo Fisher Scientific, USA) for data normalization. All experiments were performed with at least three biological replicates per experimental group (one biological replicate was defined as HDFs derived from one independent donor lot that were cultured and processed through the entire experimental workflow). For each biological replicate, more than three technical replicate wells were prepared per condition, and the measurements from these technical wells were averaged to yield a single data point per biological replicate prior to statistical analysis.

### 2.5 Mitochondrial function assessment in UVA-induced HDFs

This experiment used the same HDF cell system and replicate structure as described above for UVB (three independent donor lots, passage ≤10; three biological replicates processed on the same experimental day; three technical replicate wells for each biological replicate). The HDF cells were randomly divided into three groups (NC, UVA, and sample) and cultured in DMEM medium supplemented with 10% fetal bovine serum at 5% CO_2_ and 37 °C for 24 h. The sample group was added with 1% FGRE intervention, while the other groups received no intervention for 24 h. Then, the cells in UVA and sample groups were irradiated with UVA in PBS at a dose of 30 J/cm^2^ according to the method of Parzonko and Kiss (2019) with slight modifications. UVA were generated by a UVA lamp (Philips, PL-S 9W/10/2 P lamps) with the peak at 365 nm. The emitted radiation was checked under the microplate using a radiometer (Solar Light PMA2100, USA). After irradiation, PBS was replaced with DMEM medium, and the cells were cultured for an additional 24 h. Finally, the cells were washed with PBS 2–3 times and collected to determine the ATP content, NAD^+^/NADH ratio, and the relative expression levels of the mitochondrial genes. The ATP content and NAD^+^/NADH ratio were measured using a commercial kit (Beyotime Biotechnology, China) following the protocol of the manufacturer. The protein content was measured by commercial BCA kits for data normalization. The mRNA transcription levels of mitochondrial fusion genes (*Mfn1* and *Opa1*) and fission genes (*Drp1* and *Fis1*) were estimated using a quantitative reverse transcription polymerase chain reaction (RT-PCR) system (Bio-Rad, USA). The gene sequences of the primers are listed in Table 1. The effect of 1 μg/mL leucinic acid on ATP production in UVA-induced HDFs was determined using a fluorescent probe. Briefly, after intervention and modeling as above, the cells were washed 2–3 times with PBS, and 500 μL of 10 μM ATP-Red 1 probe was added to each well. Then, the cells were incubated at 37 °C and 5% CO_2_ for 30 min, and the probe-containing medium was removed. The cells were washed 2–3 times with PBS again, and fluorescence images were captured for subsequent acquisition and analysis. Fluorescence images were acquired with a Leica DM2500 LED microscope using the Cy3 channel at a total magnification of ×200. Three randomly selected areas were observed per sample. The ATP content was calculated as the mean integrated optical density (IOD) calculated using Image-Pro Plus 7.0 software. The mean IOD value of the NC group was used as the baseline for normalization. Specifically, the IOD value of each sample in every experimental group was divided by the mean IOD of the NC group to obtain the relative IOD (relative IOD = individual IOD/mean IOD of NC). All images were acquired under identical settings.

**TABLE 1:** Gene sequences of the primers.

Gene	Forward primer (5′-3′)	Reverse primer (5′-3′)
Mfn1	CAC​AGG​GGA​TGC​CAG​AAA​GT	TTC​ACT​GCT​GAC​TGC​GAG​AT
Opa1	ATC​ATC​TGC​CAC​GGG​TTG​TT	ATA​GGG​CCA​CAT​GGT​GAG​GA
Drp1	AGT​GGT​GAC​TTG​TCT​TCT​TCG​TAA	TAG​CCT​GTT​TCT​CCT​TTG​TTC​CT
Fis1	CCA​AGA​GCA​CGC​AGT​TTG​AG	GAC​GTA​ATC​CCG​CTG​TTC​CT

### 2.6 Non-targeted metabolomics analysis

GRE and FGRE were analyzed for non-target metabolites by Majorbio Bio-Pharm Technology (Shanghai, China). Briefly, the samples were analyzed using a Thermo UHPLC-Q Exactive HF-X system (Thermo Fisher Scientific, USA), which was equipped with an ACQUITY HSS T3 column (Waters, USA). The raw data were imported into the metabolomics processing software Progenesis QI (Waters Corporation, Milford, USA), which ultimately formed a data matrix. Subsequently, Progenesis QI 3.0 (Nonlinear Dynamics, Waters, USA) was used for feature peak library identification, matching MS and MS/MS mass spectrometry information with metabolic databases (including public and in-house databases). The identification criteria were based on the reference with modifications (Ma et al., 2026). Specifically, it included (i) mass accuracy within 10 ppm (mass error); (ii) MS/MS spectral matching score ≥35 out of 100 (fragmentation score); (iii) overall identification score ≥36 out of 60, which integrates mass accuracy (20 points), MS/MS fragment matching (20 points), and isotopic distribution matching (20 points). Level 1 compounds were verified using authentic standards with |ΔRT| ≤ 0.1 min (ΔRT = RT_sample_-RT_standard_) and met all the thresholds described above. Level 2 (putative) compounds likewise met the thresholds above and were matched against authentic standards, but they did not satisfy the full Level 1 retention-time criterion, i.e., the retention-time deviation exceeded the 0.1-min tolerance (|ΔRT| > 0.1 min) or no ΔRT value was available for comparison. Level 3 compounds with MS/MS spectral matching scores ≥40 but without authentic standards were considered putative. Pooled quality control (QC) samples were prepared by combining equal aliquots of all study samples and were injected every 10 injections throughout the analytical sequence. Blank samples were also analyzed to exclude background contaminants. Features with RSD >30% in QC samples were excluded. Data normalization was performed using probabilistic quotient normalization (PQN), with the median spectrum of QC samples serving as the reference (Tseng et al., 2025). For differential metabolite analysis, the Benjamini–Hochberg procedure was applied to control the false discovery rate (FDR) at *p-*values <0.05. The main databases used include mainstream public databases such as HMDB (http://www.hmdb.ca/), METLIN (https://metlin.scripps.edu/), KEGG (http://www.kegg.jp/kegg/kegg1.html), and Majorbio database. KEGG pathway enrichment analysis of the metabolites was performed through the Majorbio cloud platform (https://www.majorbio.com) (Kanehisa et al., 2023). For biological replicates, GRE and FGRE samples were independently prepared or fermented in 10 separate batches, respectively, according to the method in Section 2.2. Differential metabolites were identified based on FDR-*p* values, the VIP values derived from the multivariate OPLS-DA model and fold change (FC). FC was calculated as the ratio of the mean relative abundance of each metabolite in the FGRE group to that in the GRE group, and it was log2-transformed (log_2_FC) for visualization; log_2_FC > 0 indicates higher abundance in FGRE, and log_2_FC < 0 indicates higher abundance in GRE. In the primary screening, without imposing any FC cutoff, metabolites were designated as differential when all of the following criteria were satisfied: (i) FDR-*p* < 0.05 and corresponds to -log_10_ (FDR-*p*) > 1.30; (ii) VIP >1.0. This primary set was used to depict the overall differential profile (volcano plot) and for KEGG compound classification and pathway enrichment. Within this primary set, a stricter subset for heat-map analysis was defined by additionally requiring |log_2_FC| ≥ 1 (equivalent to FC ≥ 2.0 or FC ≤ 0.5) and VIP >2.0. The hierarchical clustering and heat-map visualization were performed in R-4.6.0.

### 2.7 Quantitative analysis of metabolite concentration in FGRE

The leucinic acid concentration was quantified by HPLC. The samples were analyzed using a UV detector (λ = 205 nm) in an Agilent 1260 HPLC system equipped with an XDB-C18 column (5 μm × 4.6 mm × 150 mm, with a column temperature of 30 °C). The mobile phase consisted of 0.1% (w/v) (NH_4_)_2_HPO_4_ in water(A) and acetonitrile (B), delivered at a flow rate of 1 mL/min. The gradient program was as follows: 0 min–15 min, 10% B; 15 min–20 min, 10%–20% B; 20 min–50 min, 20% B.

The samples of GRE and FGRE were pre-processed before determining the concentration of UroM5, UroM6, UroC, and UroA. A 200-µL aliquot of each sample was taken and mixed with 600 µL of acetonitrile to precipitate the proteins. After centrifugation, the supernatant was collected and evaporated to dryness. The dried samples were then redissolved in 200 µL of a 50% methanol solution and centrifuged at 12,000 rpm for 10 min. The resulting supernatant was collected for analysis using a triple quadrupole liquid chromatography–mass spectrometer (Agilent 6470, USA) equipped with Waters Acquity Premier CSH Phenyl-Hexyl (1.7 μm, 2.1 × 100 mm) (Waters, USA). The temperatures of the column and autosampler were maintained at 35 °C. The mobile phase consisted of water with 0.1% (v/v) formic acid (A) and acetonitrile with 0.1% (v/v) formic acid (B), delivered at a flow rate of 0.3 mL/min. The gradient program was as follows: 0 min–7 min, 5%–60% B; 7 min–9 min, 60%–95% B; 9 min–12 min, 95% B; 12 min–12.5 min, 95%–5% B; and 12.5 min–16.5 min, 5% B. An ion source used heated electrospray ionization (ESI) and was set as follows: dry gas flow rate, 5 L/min; dry gas temperature, 325 °C; sheath gas flow rate, 11 L/min; sheath gas temperature, 350 °C; and capillary pressure, 3,500 V. The detection used the negative ion scanning mode, with a monitoring mode of multiple reaction monitoring (MRM). Standard UroM5, UroM6, UroC, and UroA were used for qualitative and quantitative analysis.

### 2.8 Statistical analysis

The data were analyzed using one-way analysis of variance (ANOVA) with SPSS 22.0 and Origin 9.0 software. Results are presented as the mean ± standard error of the mean (SEM). Tukey’s *post hoc* test was applied following ANOVA to determine statistically significant differences (*p* < 0.05) among different groups.

## 3 Results

### 3.1 Preparation and biochemical characterization of FGRE

To ensure batch-to-batch stability, more than three batches of FGRE and GRE samples were prepared. FGRE and GRE samples were normalized to the same Brix level (7.5%) during the preparation process, and the final dry-matter was 7.01% ± 0.13% and 7.07% ± 0.09%, respectively. The mean of pH and total phenolic content among different batches of FGRE were 5.51 ± 0.07 and 353.86 ± 24.35 μg/mL, with coefficients of variation (CV) of 1.3% and 6.9%, respectively. The pH and total phenolic content in GRE were 6.65 ± 0.04 and 249.93 ± 16.42 μg/mL, with CV of 0.6% and 6.57%. These results indicated that fermentation enhanced the total phenolic content in GRE.

The biochemical experiments were used to explore the effects of FGRE on antioxidant and anti-elastase activities (Figure 1). The results demonstrated DPPH radical scavenging activity of 75.74% and elastase-inhibition of 58.12% for FGRE. Notably, FGRE exhibited significantly stronger antioxidant and anti-elastase effects than GRE (*p* < 0.05), indicating that fermentation enhances the bioactivity of GRE.

**FIGURE 1:**
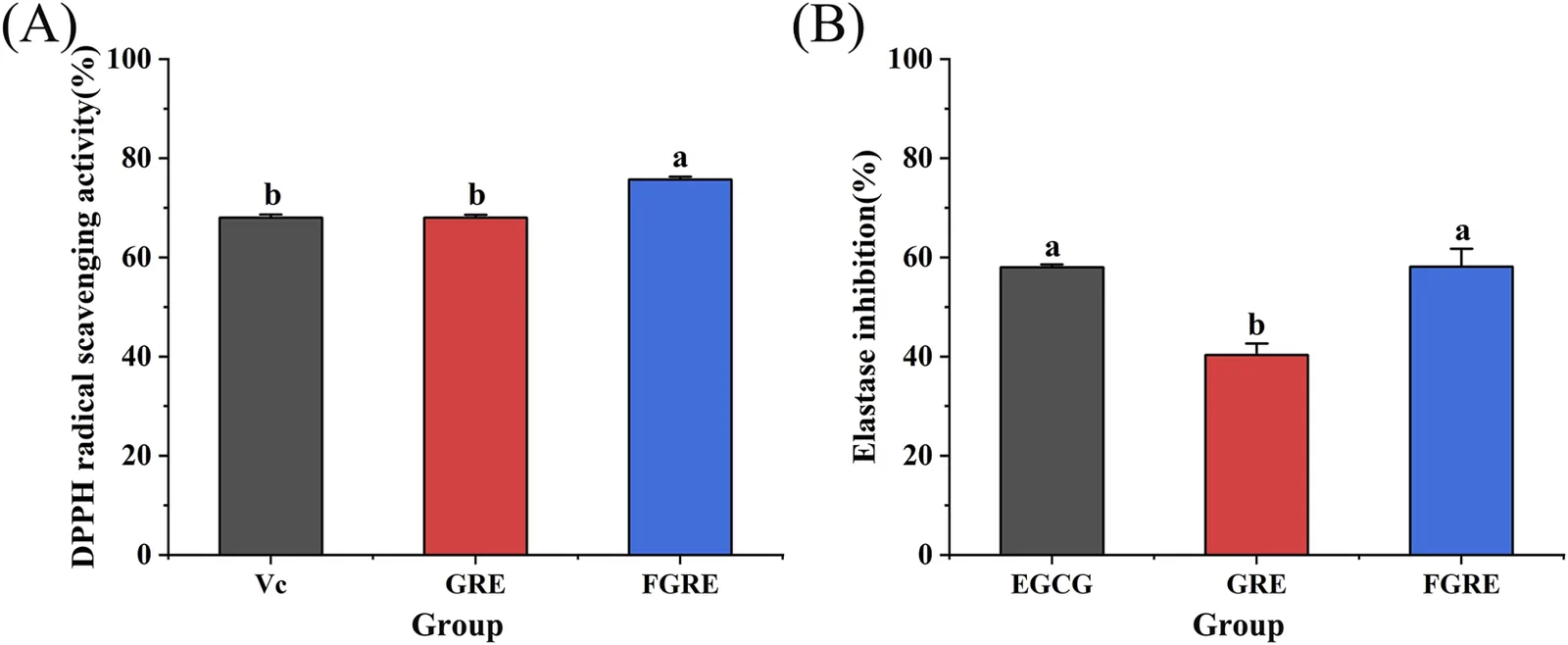
Bioactivities of FGRE determined by biochemical experiments (*n* = 3). **(A)** DPPH radical scavenging assay. **(B)** Anti-elastase activity. Letters **(A–C)** indicate significant differences among groups (*p* < 0.05): groups not sharing a letter differ significantly; groups sharing a letter are not significantly different.

### 3.2 FGRE alleviated mitochondrial respiratory disorder in UVB-induced HDF cells

The results of CCK-8 showed that different concentrations (1%–10%) of GRE and FGRE were not harmful but instead beneficial for the survival of HDF cells (Figure 2A). The effects of FGRE on mitochondrial respiratory rate of UVB-induced HDF cells were analyzed using Seahorse XF-96. As shown in Figure 2B, the OCR of the NC, GRE, and FGRE groups were markedly higher than that of the UVB group (*p* < 0.05). Compared with the NC group, the level of basal respiration, ATP production, and maximal respiration in the UVB group were markedly decreased (*p* < 0.05), indicating that UVB caused damage to cellular mitochondria (Figure 2CDE). In addition, the level of spare respiratory capacity in the NC and UVB groups were not significant (Figure 2F).

**FIGURE 2:**
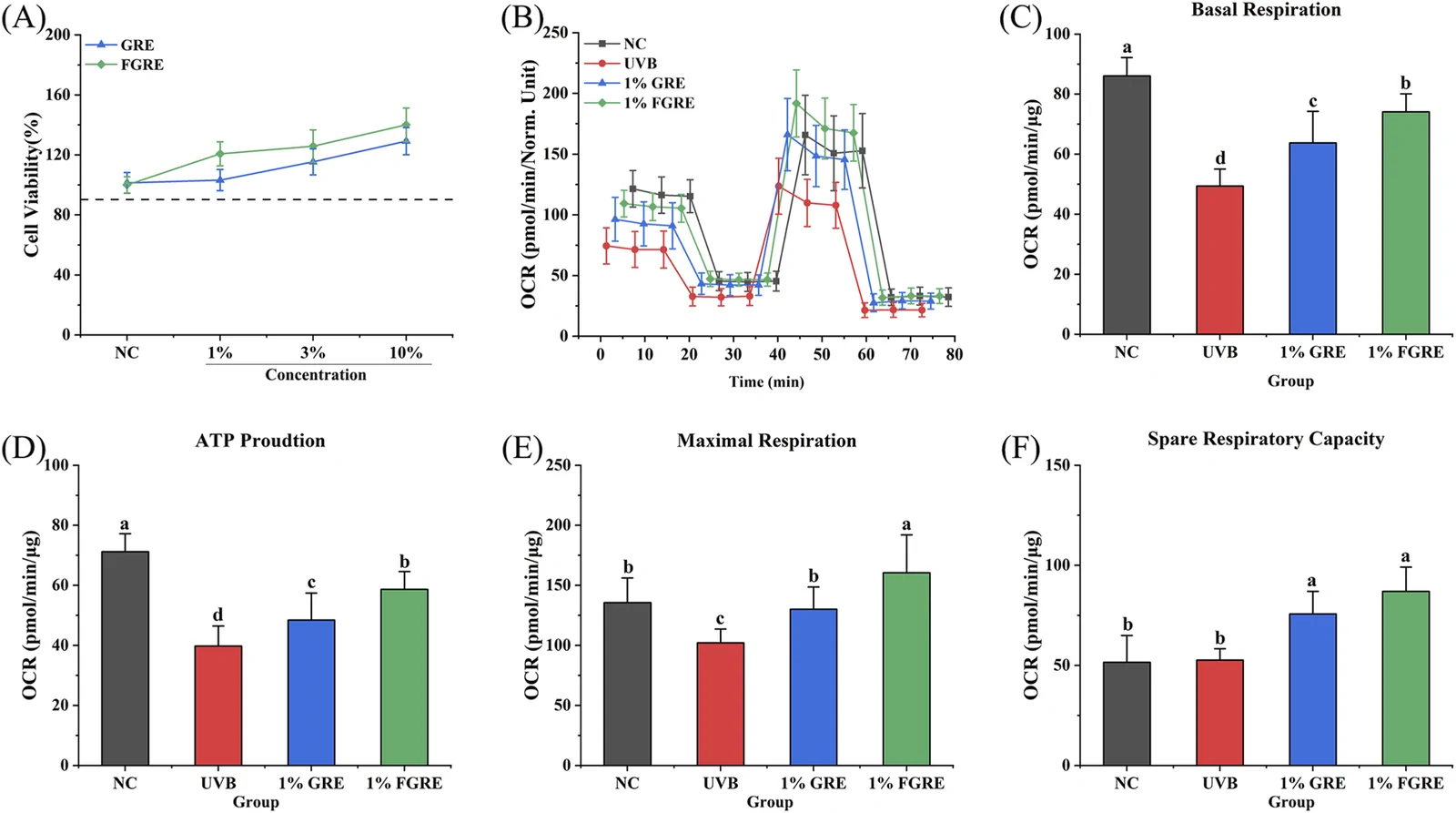
Effect of FGRE on mitochondrial respiration in UVB-induced HDF cells (*n* = 3). **(A)** Cell viability. **(B)** Cellular OCR curve. **(C)** Basal respiration. **(D)** ATP production. **(E)** Maximal respiration. **(F)** Spare respiration capacity. Letters **(A–C)** indicate significant differences among groups (*p* < 0.05): groups not sharing a letter differ significantly; groups sharing a letter are not significantly different.

According to Supplementary Figures S1, 1%–10% FGRE significantly improved the level of cellular basal respiration, ATP production, maximal respiration, and spare respiratory capacity compared to that in the UVB group (*p* < 0.05). This means that 1% FGRE was sufficient to mitigate cellular mitochondrial respiratory disorder caused by UVB irradiation. Consequently, compared to the difference in effect at the same concentration (1%) between FGRE and GRE, the results indicated that both GRE and FGRE intervention effectively enhanced the indicators related to mitochondrial respiratory metabolism compared to that in the model group (*p* < 0.05). Particularly, the FGRE group was significantly more effective than the GRE group on cellular basal respiration, ATP production, and maximal respiration (*p* < 0.05). These results indicated that FGRE could ameliorate the UVB-induced impairment of cellular mitochondrial function better.

### 3.3 FGRE relieved mitochondrial dysfunction in UVA-induced HDF cells

Given the protective effects observed in the UVB model, we next explored whether FGRE could also mitigate UVA-induced damage by regulating mitochondrial homeostasis (Figure 3). The results showed that 1% FGRE markedly increased the ATP content and NAD^+^/NADH ratio compared with that in the UVA group (*p* < 0.05), indicating that FGRE was beneficial in protecting the cellular mitochondrial function disorder.

**FIGURE 3:**
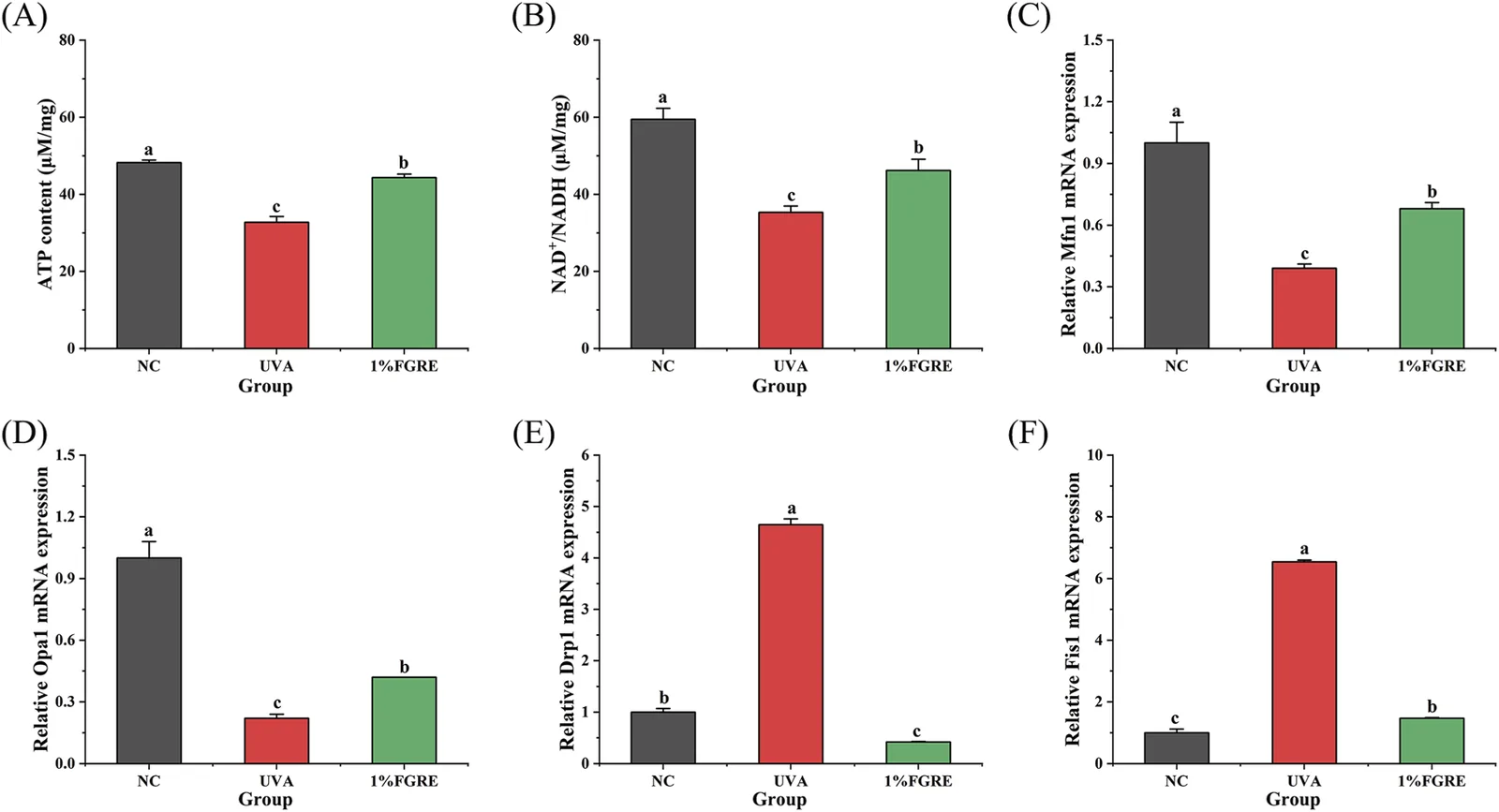
Effect of FGRE on mitochondrial function in UVA-induced HDF cells (*n* = 3). **(A)**ATP content. **(B)** NAD^+^/NADH ratio. **(C)** Relative *Mfn1* mRNA expression. **(D)** Relative *Opa1* mRNA expression. **(E)** Relative *Drp1* mRNA expression. **(F)** Relative *Fis1* mRNA expression. Letters **(A–C)** indicate significant differences among groups (*p* < 0.05): groups not sharing a letter differ significantly; groups sharing a letter are not significantly different.

The mitochondrial quality control (MQC) is crucial for mitochondrial functional homeostasis, and the process of mitochondrial fusion and fission is one of the quality control mechanisms. Thus, the relative expression of fusion and fission genes was measured. With UVA irradiation, the transcription levels of *Mfn1* and *Opa1* in HDF cells sharply declined, while the transcription levels of *Drp1* and *Fis1* markedly upregulated (*p* < 0.05) compared to that in cells with no UVA exposure. The results indicated UVA exposure disturbed mitochondrial homeostasis. Subsequently, 1% FGRE intervention relieved the imbalance. Compared to that in the UVA group, FGRE significantly elevated the relative expression of fusion genes and inhibited the relative expression of fission genes (*p* < 0.05). All these results showed that FGRE regulated the expression of mitochondrial dynamics-related genes in UVA-induced HDFs.

### 3.4 Non-targeted metabolomics analysis of FGRE

In order to explore the potentially active metabolites in FGRE, the non-targeted metabolomics analysis of GER and FGRE were performed. The principal component analysis (PCA) of GER and FGRE is presented in Figure 4A. The contribution of the first principal component is 68.30%, while the contribution of the second principal component is 4.23%. The first principal component explained 68.30% of the variance, whereas the second principal component accounted for only 4.23%. Along this first principal component, FGRE and GRE were clearly separated, with FGRE positioned far from GRE. The orthogonal partial least squares discriminant analysis (OPLS-DA) model was established for further analysis. The scores plot (Figure 4B) revealed a clear separation trend between the two groups along the predictive component axis (t [1] = 83.10%), with all samples located within the 95% Hotelling’s T^2^ confidence ellipses. These results indicate a significant difference in the metabolic profiles between FGRE and GRE. Based on the FDR-*p-*values (*p* < 0.05, which corresponds to -log_10_FRP-*p* > 1.30) and the VIP values (VIP >1), a total of 687 differential metabolites were preliminary identified (Figure 4C). There were 236 significant upregulated metabolites and 451 significant downregulated metabolites on comparing FGRE and GRE. For further research, the compounds classification and KEGG enrichment of differential metabolites between FGRE and GRE were analyzed (Figure 4DE). The metabolites were divided into 15 categories, and most metabolites concentrated on amino acids and carboxylic acids. Furthermore, KEGG enrichment analysis indicated that all differential metabolites were enriched in 20 metabolic pathways, and most were enriched in the ABC transporters pathway and biosynthesis of cofactors pathway. A few differential metabolites concentrated on metabolic pathways, including aminoacyl-tRNA biosynthesis, nucleotide metabolism, D-amino-acid metabolism, and purine metabolism. The rich factor of valine, leucine, and isoleucine biosynthesis was the maximum in all pathways, indicating that changes in valine, leucine, and isoleucine biosynthesis maybe caused by fermentation.

**FIGURE 4:**
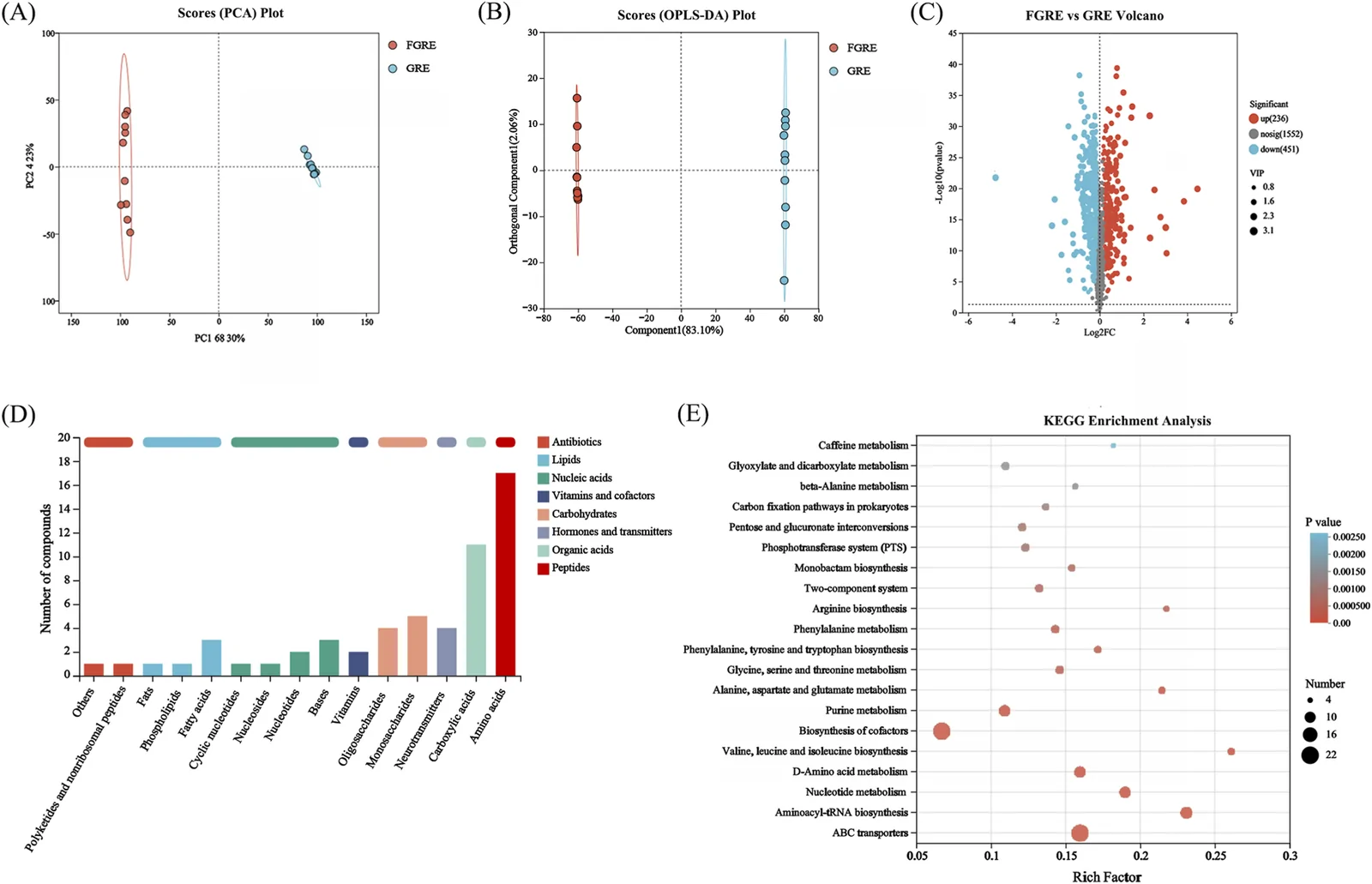
Non-targeted metabolomics analysis of FGRE and GRE (*n* = 10). **(A)** PCA analysis. **(B)** OPLS-DA analysis. **(C)** Volcano plot. **(D)** Compounds classification. **(E)** KEGG enrichment analysis. **(E)** Heatmap of differential metabolites.

Subsequently, a stricter screening was applied with thresholds of |log_2_FC| ≥ 1 (corresponding to FC ≥ 2 or FC ≤ 0.5), FDR-*p* < 0.05, and VIP >2. After manual verification combined with reference, a final set of 24 differential metabolites was obtained (Figure 5; Supplementary Table S2). Specifically, 11 metabolites were significantly increased and 13 were significantly decreased among the identified differential metabolites. The obvious reduced metabolites of FGRE were mainly amino acids and small-molecule peptides, such as some tripeptides (Leu-Ser-Ile, Ile-Asn-Val, and His-Gly-Glu), some dipeptides (Ala-Ile, Ser-Ile, Ala-Leu, and Thr-Ile), and some amino acids (L-arogenate, L-glutamic acid, and L-aspartic acid). In addition, choline glycerophosphate (putatively from yeast extract in the culture medium) and afzelechin (a phenolic acid of potential plant origin) were also significantly decreased. The composition of upregulated metabolites in FGRE was quite complex. The metabolites chakanoside I, cyclokievitone, and 6-geranylnaringenin belong to flavans and isoflavans, which were significantly increased in the FGRE group. Some amino acid derivatives and peptides were increased after fermentation, such as leucinic acid, Val-Asn, and Arg-Val-Phe. In addition, UroA and UroM5 were also markedly increased in FGRE, which might be the special metabolites in *G. rhois* fermented by *L. plantarum*. There were additional changes in other microbial metabolites, such as riboflavin reduced and 2-pentanone increased.

**FIGURE 5:**
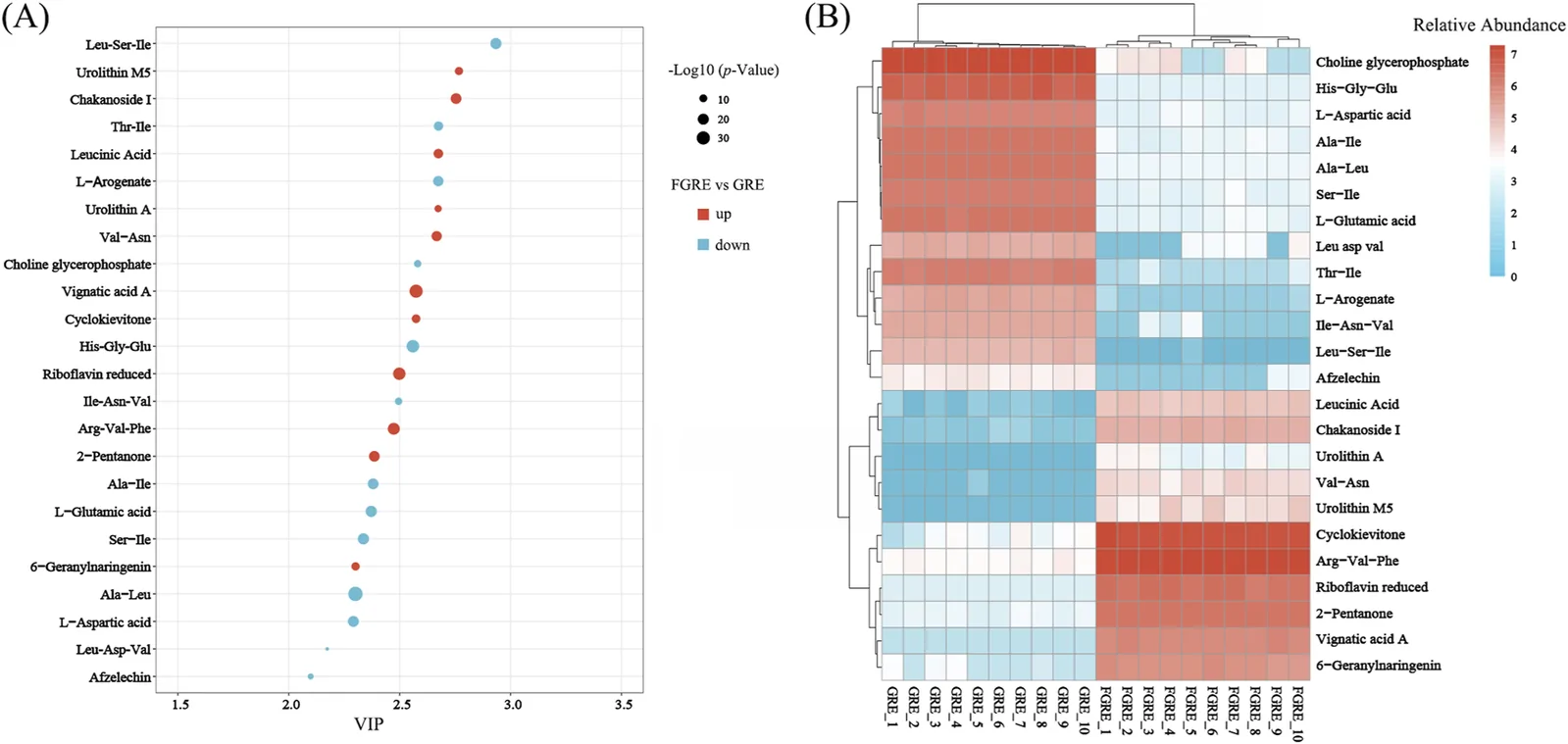
VIP value **(A)** and Heatmap **(B)** of 24 differential metabolites (*n* = 10).

### 3.5 Quantitative analysis of metabolites in FGRE

To explore the potential active metabolites in FGRE, metabolites (UroA, UroM5, and leucinic acid) exhibiting high VIP values and marked increases in abundance were subjected to both qualitative annotation and quantitative determination (Table 2). Since no reference standard of chakanoside I was available, this metabolite was not pursued in subsequent experiments. The results showed that GRE contained 0.01 ng/mL UroM5 and 26.96 μg/mL leucinic acid, while no UroA was detected. In contrast, the levels of UroA, UroM5, and leucinic acid all increased in FGRE after fermentation; they increased to 0.49 ng/mL, 8.35 ng/mL, and 97.20 μg/mL, respectively.

**TABLE 2:** Concentration of metabolites in FGRE and GRE (*n* = 3).

Compounds	Qualitative analysis			Quantitative analysis	
	Mode	Adducts	Fragments	Content in GRE	Content in FGRE
UroM5	Negative	[M-H]^-^	275.0178, 256.9908, 228.9958	0.01 ± 0.01^b^ ng/mL	8.35 ± 0.93^a^ ng/mL
UroA	Negative	[M-H]^-^	227.0335, 198.0410, 182.0450, 171.0244	-	0.49 ± 0.06 ng/mL
Leucinic acid	Negative	[2 M-H]^-^	263.0522, 191.0364, 131.0715, 85.0661	26.96 ± 0.60^b^ μg/mL	97.20 ± 1.88^a^ μg/mL

^a^ There was statistical significance (*p* < 0.05) between GRE and FGRE.

^b^ There was statistical significance (*p* < 0.05) between GRE and FGRE.

These results indicated that the fermentation process involved the transformation of substances. Given the limitations of untargeted metabolomic analysis, we also analyzed the possible presence of other UroA derivatives with the standard. Unfortunately, UroC and UroM6 were not detected in either GER or FGRE.

### 3.6 Leucinic acid alleviated mitochondrial dysfunction in HDF cells

The effect of leucinic acid, the differential metabolite in FGRE, was explored on cellular mitochondrial function (Figure 6). The results of CCK-8 presented that the different levels of leucinic acid below 30 μg/mL were non-toxic to HDF cells, and 0.3 μg/mL–3 μg/mL leucinic acid markedly increased the cell viability (Figure 6A). The effect of different concentrations of leucinic acid on the mitochondrial respiratory capacity in UVB-induced HDF cells was dose-dependent. The effect of 0.2 μg/mL leucinic acid was not significant in the UVB group. In contrast, 1 μg/mL–5 μg/mL leucinic acid markedly increased the OCR of cellular basal respiration, ATP production, and maximal respiration compared to that in the UVB group (*p* < 0.05). These results indicated that 1 μg/mL–5 μg/mL leucinic acid could improve the mitochondrial respiratory metabolism in UVB-induced HDF cells.

**FIGURE 6:**
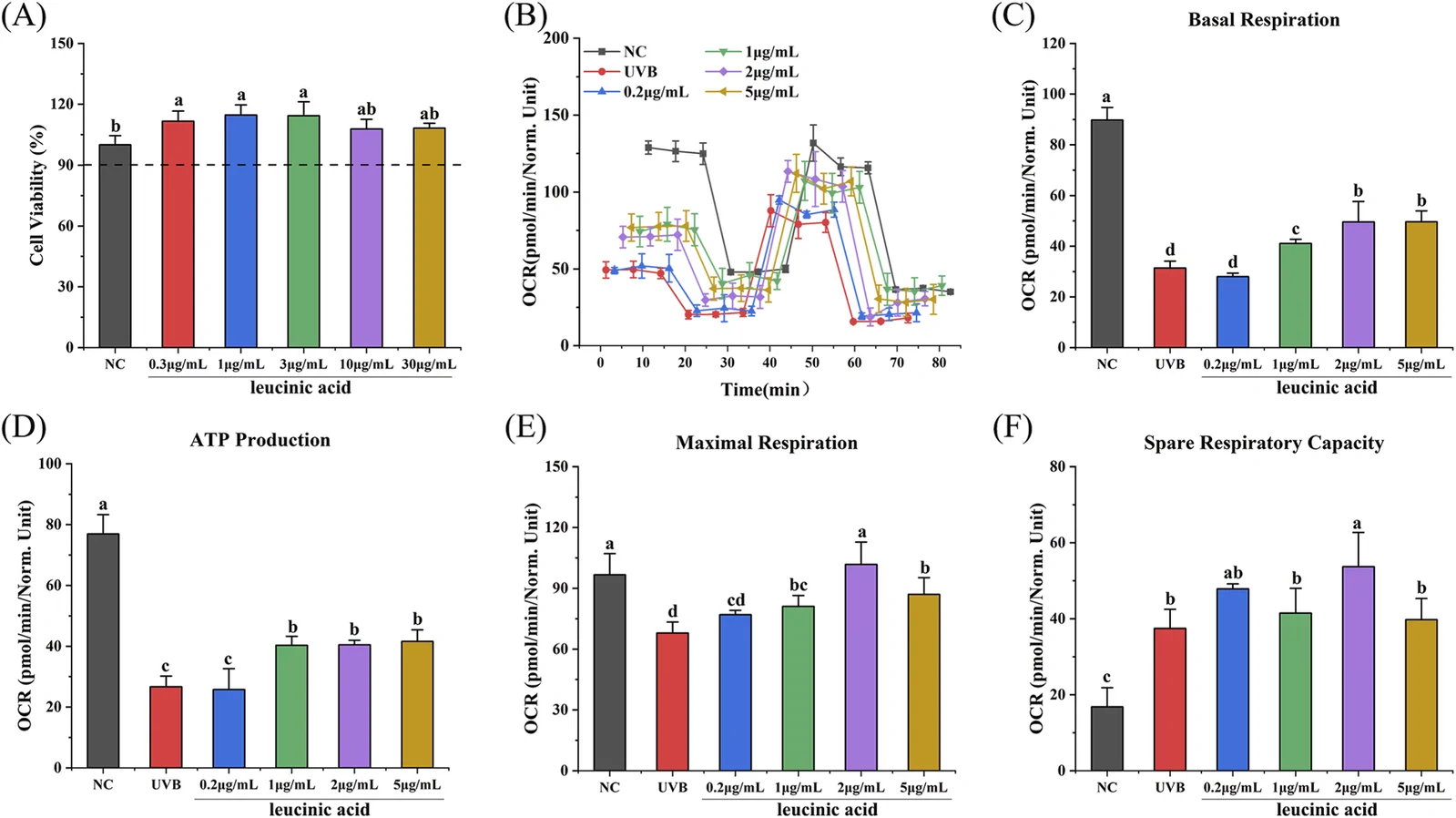
Effect of leucinic acid on mitochondrial respiratory in UVB-induced HDF cells (*n* = 3). **(A)** Cell viability. **(B)** Cellular OCR curve. **(C)** Basal respiration. **(D)** ATP production. **(E)** Maximal respiration. **(F)** Spare respiration capacity. Letters **(A–D)** indicate significant differences among groups (*p* < 0.05): groups not sharing a letter differ significantly; groups sharing a letter are not significantly different.

Based on the findings from the UVB model, we further explored the effect of 1 μg/mL leucinic acid on ATP production in UVA-induced HDF cells. As shown in Figure 7, the relative mean IOD of ATP in cells treated with leucinic acid was observed to be significantly higher than that in the UVA group (*p* < 0.05), which also was close to that of the NC group. The results indicated that 1 μg/mL leucinic acid mitigated mitochondrial dysfunction in UV-stimulated HDF cells. In addition, 1 μg/mL leucinic acid was equivalent to the amount present in 1% FGRE, indicating that leucinic acid was likely to be the potentially active metabolite in FGRE.

**FIGURE 7:**
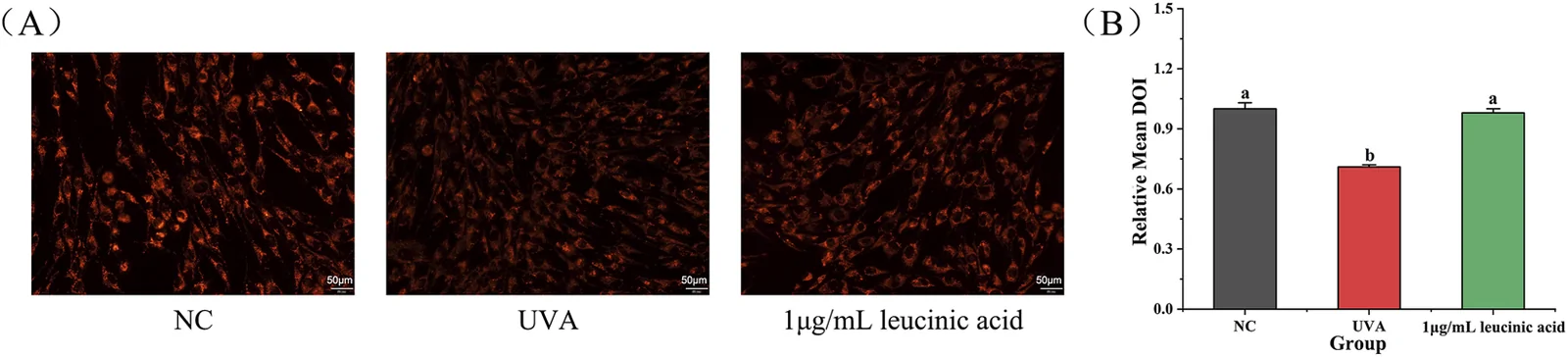
Effect of leucinic acid on ATP production in UVA-induced HDFs (*n* = 3). **(A)** Fluorescence image magnified ×200, scale bar value 50 μm. **(B)** Mean IOD of ATP. Letters **(A–C)** indicate significant differences among groups (*p* < 0.05): groups not sharing a letter differ significantly; groups sharing a letter are not significantly different.

## 4 Discussion

*G. rhois* is primarily applied externally in clinical practice for burn management and tissue repair facilitation, in addition to its oral administration as a traditional Chinese medicinal agent. Although microbial fermentation is widely utilized in modifying traditional Chinese medicinal materials, the application of *Lactobacillus* strains for biotransformation of GRE to potentiate their bioactive potential remains underexplored in pharmacological research. Thus, we fermented and obtained FGRE by *L. plantarum*. Experimental analysis demonstrated that FGRE had higher antioxidant and anti-aging activities than GRE *in vitro*, and it significantly enhanced the mitochondrial respiratory capacity in UVB-induced HDF cells. These findings indicated that fermentation indeed enhanced the biological activity.

As a high-turnover organ, the skin’s continuous cellular renewal and proliferation depend on mitochondrial respiration to meet its energy demands. However, in addition to chronological aging (time-induced), photoaging caused by UV radiation is a major cause mitochondrial dysfunction (Zhang et al., 2023a). The radiation that reaches human skin is composed primarily of UVB (290 nm–320 nm) and UVA (320 nm–400 nm) (Gromkowska-Kępka et al., 2021). UVB mainly affects the epidermis and superficial dermis, while UVA penetrates both the epidermis and the entire dermis (Guan et al., 2021). Numerous studies indicate that both UVA and UVB radiation impair mitochondrial homeostasis and function, manifesting as mitochondrial DNA damage, overproduction of mitochondrial ROS, dysregulation of apoptotic pathways, and metabolic disturbances, which contribute to cutaneous photodamage, photoaging, and even skin carcinogenesis (Yuan et al., 2025). Thus, we evaluated the effects of UVB and UVA irradiation on mitochondrial function in HDF cells. Zheng et al. (2025) stated that the OCR of basal respiration, maximal respiration, and ATP production in UVB-induced HaCaT keratinocytes were significantly lower than that in the untreated group by Seahorse XF. In our study, we found similar results in HDF cells, and we compared the effect of FGRE and GRE. FGRE presented the better effect on mitochondrial respiration than unfermented GRE, which also indicated the enhancement of bioactivity by fermentation. Given the protective effects observed in the UVB model, we next explored whether the fermented product could also mitigate UVA-induced damage by regulating mitochondrial homeostasis. Mitochondrial dysfunction is a multifactorial phenotype encompassing bioenergetic failure, redox imbalance, and disrupted dynamics (Quan et al., 2025). UVA irradiation decreased ATP content and suppressed the NAD^+^/NADH ratio in HDFs (Ye et al., 2026), which was consistent with our experimental findings. FGRE markedly increased ATP generation and upregulated the ratio of NAD^+^/NADH in UVA-induced cells, indicating the protective effects on maintaining mitochondrial bioenergetic activity and redox state.

Mitochondria maintain healthy structure and function through the homeostasis of mitochondrial dynamics, particularly the balance between fission and fusion (Quan et al., 2025). Mitochondrial fusion occurs in two steps: MFN1/2-mediated outer membrane fusion is regulated, and OPA1-dependent inner membrane fusion is coordinated (Chandhok et al., 2018). When cellular ATP-demand increases, enhanced mitochondrial fusion merges individual organelles to stabilize the internal environment, thereby improving energy production efficiency. Meanwhile, fusion expands the mitochondrial matrix volume to facilitate metabolite exchange and preserves both bioenergetic efficiency and organellar integrity in the face of oxidative stress (Chen et al., 2023; Quintana-Cabrera and Scorrano, 2023). DRP1 and FIS1 are involved in the regulation of mitochondrial fission. Mitochondrial fission leads to morphological fragmentation and is associated with mitochondrial uncoupling and permits segregation of dysfunctional mitochondria to become targets of mitochondrial autophagy (Gu et al., 2021). Recent evidence has demonstrated that the activities of the above-mentioned fusion and fission proteins are tightly governed by a variety of upstream signaling pathways. The AMPK/SIRT1/PGC-1 signaling pathway serves as a critical hub integrating cellular energy status, epigenetic regulation, and transcriptional programs (Wang et al., 2026). AMPK and SIRT1 as two pivotal cellular energy sensors and cooperate to jointly activate PGC-1α. Activated PGC-1α not only drives mitochondrial biogenesis but also orchestrates mitochondrial dynamics through the modulation of fusion and fission genes (Auwerx and Cantó, 2009). Dysregulation between fusion and fission accelerates cellular senescence, characterized by metabolic disturbances, oxidative stress, apoptosis, and related processes (Wu et al., 2026). UVA-induced aging skews the equilibrium of mitochondrial dynamics toward excessive fission and impaired fusion, leading to mitochondrial fragmentation and dysfunction (Niccolini et al., 2025). In addition, polydatin has been demonstrated to mitigate UVA-induced mitochondrial fission and lower oxidative stress in UVA-induced HDFs (Niccolini et al., 2025). In addition, Gupta et al. also found that UVB led to mitochondrial fission mediated by the induction of Drp-1, and rosmarinic acid alleviated UVB-induced photodamage (endoplasmic reticulum stress and mitochondrial dysfunction) in HDFs and mice (Gupta et al., 2023). In this study, FGRE prevented the UVA-induced increase the relative expression of *Drp1* and *Fis1* and the decrease of the relative expression of *Mfn1* and *Opa1*. Thus, FGRE may alleviate mitochondrial dysfunction caused by UV though regulating mitochondrial dynamics-related genes. This speculation required further evaluation of the effects of FGRE on UV-induced mitochondrial dysfunction at the protein level and in terms of mitochondrial morphology, membrane potential, and other dimensions. In addition, the type of UV radiation should also be taken into consideration when exploring these dimensions.

To explore the material basis for the efficacy of FGRE, we conducted a metabolomics analysis between FGRE and GRE. The main distinguishing metabolites comprise short peptides and free-form amino acids. Studies demonstrated that *L*. *plantarum* fermentation of milk or milk protein generated differential metabolites, which were predominantly short peptides and bioactive amino acids (Mazzei and Piccolo, 2012). Meanwhile, the increased concentration of amino acids not only promotes strain growth but also enhances nutritional value and functional efficacy (Wang et al., 2019). KEGG enrichment analysis revealed that fermentation processes significantly modulated the branched-chain amino acids (BCAAs, including valine, leucine, and isoleucine) biosynthesis pathway. Supplementation with branched-chain amino acids is generally beneficial for regulating lifespan, as demonstrated in worms, flies, and rodents (Li and Hoppe, 2023). This is because the degradation of branched-chain amino acids primarily occurs in the mitochondrial matrix, where it participates in energy metabolism, mitochondrial biogenesis, and protein quality control in both the mitochondria and cytosol. These age-related effects of amino acids are achieved through the direct or indirect regulation of mitochondrial function (Li and Hoppe, 2023). Leucinic acid is considered the end metabolite of leucine, which can be converted from leucine to α-ketoisocaproic acid (α-KIC) and further converted from α-KIC. This transformation can be conducted by *Lactococcus* with aminotransferase and hydroxyacid dehydrogenase (Chambellon et al., 2009). Thus, the increase of leucinic acid in FGRE is due to the fermentation by *L*. *plantarum*. Leucinic acid has been reported to exhibit antibacterial properties and promote muscle growth (Mero et al., 2010; Pahalagedara et al., 2022), but few studies have analyzed its effects on mitochondrial function. According to our results, leucinic acid also was found to enhance mitochondrial energy metabolism in UV-induced HDF cells. Moreover, 1 μg/mL leucinic acid (equivalent to the concentration in 1% FGRE) ameliorated the mitochondrial respiratory dysfunction in HDF cells, indicating that leucinic acid was considered the potential effector in FGRE. To establish whether leucinic acid is indeed the key active metabolites responsible for FGRE activity, it is necessary to conduct experiments involving depletion, supplementation, or reconstitution of the metabolite. In addition, we conducted molecular docking between leucinic acid and human lactate dehydrogenase including human D-lactate dehydrogenase (hLDHD) and LDHA subunit (hLDHA) (Supplementary Figure S3). The results showed that the docking scores were −8.76 and −7.33, with the binding free energy of −52.16 kcal/mol and −128.29 kcal/mol, respectively. These results indicate that hLDHD or hLDHA may potentially participate in the conversion of L-leucinic acid. Based on literature references, we hypothesize that leucinic acid may be converted into α-KIC through a reversible reaction, and α-KIC can further be converted into acetyl-CoA, which can then participate in the TCA cycle to regulate cellular energy metabolism (Sivanand and Vander Heiden, 2020).

Studies have shown that the Lactobacillaceae possesses various enzymes capable of transforming phenolic compounds derived from plants or foods, such as esterase, decarboxylase, and glycosyl hydrolase, among others (Gaur and Gänzle, 2023). Given the abundance of ellagic acid in GRE, our initial strain selection was informed by literature data and experimental evaluation of their ellagic acid metabolism. *L. plantarum*, *Bifidobacterium pseudocatenulatum*, *Streptococcus thermophilus*, and related gut microbiota have been shown to transform either ellagitannins or ellagic acid into UroA through microbial fermentation (Zhang et al., 2023b). Furthermore, the *lac1563* gene was identified as a lactonase gene responsible for converting ellagic acid to urolithin A in *Limosilactobacillus fermentum* FUA033, as demonstrated by gene knockout and heterologous expression assays (Wang et al., 2025). Therefore, we fermented multiple strains of *Lactobacillus* in MRS medium containing ellagic acid and screened for strain *L. plantarum* PIAS240228 based on its conversion efficiency of ellagic acid to urolithin A (data not shown). This strain was subsequently used for GRE fermentation. The presence of UroM5 and UroA in FGRE indicated that ellagic acid in GRE was biotransformed into UroM5 and further converted into UroA by a series of dehydroxylation reactions by *L*. *plantarum* (Banc et al., 2023). The undetectable levels of metabolic intermediate UroM6 and UroC likely reflect sub-threshold metabolite concentrations rather than non-occurrence of the reaction. Given that this conversion depends on substrates, bacterial strains, and fermentation conditions (such as pH and time), compound standardization is necessary. UroA has been reported to induce mitophagy and stimulate mitochondrial biogenesis across different cell lines, typically ranging from 0.1 to 20 µM (0.023 μg/mL–4.56 μg/mL), while the bioactivity profile of its structural analog UroM5 remains largely unexplored in mitochondrial contexts (Ryu et al., 2016; Esselun et al., 2021; Liu et al., 2022; Faitg et al., 2024). According to Supplementary Figure S4, the effects of different concentrations of UroA (0.03 μg/mL–0.1 μg/mL) and UroM5 (0.3 μg/mL–1 μg/mL) on cellular respiratory metabolism under UVB-induced HDFs were analyzed. Among the tested conditions, only UroA at 0.03 μg/mL showed significant differences in both ATP production and basal respiration compared with that in the UVB-irradiated group, whereas UroM5 at 0.3 μg/mL significantly increased the basal respiration production with no significant effects on the other measured parameters. Therefore, it was initially speculated that the tested concentrations of UroA and UroM5 were below their effective thresholds, and the mitochondrial respiratory benefit of FGRE may be attributed primarily to leucinic acid. Furthermore, we explored the influence of extremely low levels of UroA (0.005 μg/mL–0.025 ng/mL) and UroM5 (0.08 μg/mL–0.4 ng/mL) on ROS production in UVB-induced HaCaT cells. Interestingly, we found that both UroA and UroM5 suppressed ROS production, and this effect was also observed at equivalent concentrations (0.005 ng/mL UroA and 0.08 ng/mL UroM5) of 1% FGRE (Supplementary Figure S5). The results confirmed their antioxidant effect. In addition, these results indicated new possibilities that require further validation: (i) whether lower concentration of UroA (below 0.03 μg/mL) and UroM5 (below 0.3 μg/mL) can improve mitochondrial function; (ii) synergistic interactions between UroA and UroM5, or among them and leucinic acid due to complex composition of FGRE. Thus, further experiments are needed to validate these hypotheses, such as by examining the individual and combined effects of UroA and UroM5 at equivalent concentrations on mitochondrial function. Based on these results, the observed differences in bioactivity may be partially attributable to bacterial metabolites. The addition of a bacterial fermentation control (medium + strain, without GRE) may further support the activity attributed to GRE biotransformation.

Our study identified FGRE as a promising candidate for improving mitochondrial function in UV-induced HDFs, albeit with certain limitations. While our *in vitro* data are compelling, *in vivo* studies using skin equivalents or animal models are necessary to confirm the translational potential. Furthermore, given the central role of mitochondrial dysfunction in skin aging, FGRE may be developed as a functional ingredient in topical formulations or nutraceuticals aimed at preventing or mitigating UV-induced skin damage. Moreover, the increasing consumer demand for natural and fermented metabolites in cosmetics and dermatological products aligns well with the profile of FGRE. Future research should focus on (i) optimizing the fermentation conditions to increase the yield of bioactive metabolites, (ii) formulating FGRE into stable topical vehicles with appropriate skin-penetration profiles, and (iii) conducting clinical trials to evaluate its efficacy in reducing clinical signs of photoaging, such as wrinkle depth, skin laxity, and pigmentation. Additionally, safety assessments, including skin irritation and sensitization tests, will be essential prior to commercial application.

## 5 Conclusion

In this study, we fermented GRE using *L. plantarum* and found that FGRE had better antioxidant and anti-elastase activities than GRE through biochemical experiments. The obtained FGRE was also proven to enhance the mitochondrial respiratory metabolism and regulate the mRNA expression related to mitochondrial dynamics in UV-induced HDF cells. Combined with metabolomics analysis, FGRE was found to contain leucinic acid, UroM5, and UroA. In addition, mitochondrial function-enhancing effects of leucinic acid were confirmed by cellular experiments at concentrations equivalent to those in FGRE, and leucinic acid was consequently identified as the potential bioactive metabolite in FGRE. Therefore, FGRE with bioactive metabolites establishes a novel solution for the prevention of acute UV-induced mitochondrial dysfunction.

## Data Availability

The original contributions presented in the study are included in the article/Supplementary Material; further inquiries can be directed to the corresponding authors.

## References

[B1] Auwerx J. Cantó C. (2009). PGC-1α, SIRT1 and AMPK, an energy sensing network that controls energy expenditure. Curr. Opin. Lipidol. 20 (2), 98–105. 10.1097/MOL.0b013e328328d0a4 19276888 PMC3627054

[B2] Banc R. Rusu M. E. Filip L. Popa D.-S. (2023). The impact of ellagitannins and their metabolites through gut microbiome on the gut health and brain wellness within the gut–brain axis. Foods 12 (2), 270. 10.3390/foods12020270 36673365 PMC9858309

[B3] Chambellon E. Rijnen L. Lorquet F. Gitton C. van Hylckama Vlieg J. E. Wouters J. A. (2009). The D-2-hydroxyacid dehydrogenase incorrectly annotated PanE is the sole reduction system for branched-chain 2-keto acids in Lactococcus lactis. J. Bacteriol. 191 (3), 873–881. 10.1128/jb.01114-08 19047348 PMC2632062

[B4] Chandhok G. Lazarou M. Neumann B. (2018). Structure, function, and regulation of mitofusin-2 in health and disease. Biol. Rev. 93 (2), 933–949. 10.1111/brv.12378 29068134 PMC6446723

[B5] Chen W. Zhao H. Li Y. (2023). Mitochondrial dynamics in health and disease: mechanisms and potential targets. Signal Transduct. Target. Ther. 8 (1), 333. 10.1038/s41392-023-01547-9 37669960 PMC10480456

[B6] Chinese Pharmacopoeia Commission (2022). Pharmacopoeia of the People's Republic of China. Beijing: China Medical Science and Technology Press.

[B7] Commander S. Chang D. Fakhro A. Nigro M. Lee E. (2016). Noninvasive facial rejuvenation. Part 1: patient-directed. Seminars Plastic Surg. 30, 129–133. 10.1055/s-0036-1584820 27478421 PMC4961507

[B8] Djakpo O. Yao W. (2010). Rhus chinensis and galla Chinensis--folklore to modern evidence: review. Phytother. Res. 24 (12), 1739–1747. 10.1002/ptr.3215 20564459 PMC7167973

[B9] Esselun C. Theyssen E. Eckert G. P. (2021). Effects of urolithin A on mitochondrial parameters in a cellular model of early alzheimer disease. Int. J. Mol. Sci. 22 (15), 8333. [Online]. 10.3390/ijms22158333 34361099 PMC8347929

[B10] Faitg J. D’Amico D. Rinsch C. Singh A. (2024). Mitophagy activation by urolithin A to target muscle aging. Calcif. Tissue Int. 114 (1), 53–59. 10.1007/s00223-023-01145-5 37925671 PMC10791945

[B11] Frank R. Bryan C. Ulrich B. Robert H. (2006). Dred microorganism cultures and method for producing same. U.S. Patent No 7,037,708. Washington, DC: U.S. Patent and Trademark Office.

[B12] Fu H. Zhang Y. An Q. Wang D. You S. Zhao D. (2022). Anti-photoaging effect of Rhodiola rosea fermented by Lactobacillus plantarum on UVA-damaged fibroblasts. Nutrients 14 (11), 2324. 10.3390/nu14112324 35684124 PMC9183149

[B13] Gaur G. Gänzle M. G. (2023). Conversion of (poly)phenolic compounds in food fermentations by lactic acid bacteria: novel insights into metabolic pathways and functional metabolites. Curr. Res. Food Sci. 6, 100448. 10.1016/j.crfs.2023.100448 36713641 PMC9876838

[B14] Go J. Kim J. E. Koh E. K. Song S. H. Kang H. G. Lee Y. H. (2017). Hepatoprotective effect of gallotannin-Enriched extract isolated from gall on hydrogen peroxide-induced cytotoxicity in HepG2 cells. Pharmacogn. Mag. 13 (Suppl. 2), S294–s300. 10.4103/pm.pm_424_15 28808395 PMC5538169

[B15] Gromkowska-Kępka K. J. Puścion-Jakubik A. Markiewicz-Żukowska R. Socha K. (2021). The impact of ultraviolet radiation on skin photoaging - review of in vitro studies. J. Cosmet. Dermatol 20 (11), 3427–3431. 10.1111/jocd.14033 33655657 PMC8597149

[B16] Gu Y. Han J. Jiang C. Zhang Y. (2020). Biomarkers, oxidative stress and autophagy in skin aging. Ageing Res. Rev. 59, 101036. 10.1016/j.arr.2020.101036 32105850

[B17] Gu H. Yang K. Wu Q. Shen Z. Li X. Sun C. (2021). A link between protein acetylation and mitochondrial dynamics under energy metabolism: a comprehensive overview. J. Cell. Physiology 236 (12), 7926–7937. 10.1002/jcp.30461 34101176

[B18] Guan L. L. Lim H. W. Mohammad T. F. (2021). Sunscreens and photoaging: a review of current literature. Am. J. Clin. Dermatol 22 (6), 819–828. 10.1007/s40257-021-00632-5 34387824 PMC8361399

[B19] Gupta D. Sharma R. R. Rashid H. Bhat A. M. Tanveer M. A. Abdullah S. T. (2023). Rosmarinic acid alleviates ultraviolet-mediated skin aging *via* attenuation of mitochondrial and ER stress responses. Exp. Dermatol. 32 (6), 799–807. 10.1111/exd.14773 36811401

[B20] Jo C. S. Myung C. H. Yoon Y. C. Ahn B. H. Min J. W. Seo W. S. (2022). The effect of Lactobacillus plantarum extracellular vesicles from Korean women in their 20s on skin aging. Curr. Issues Mol. Biol. 44 (2), 526–540. 10.3390/cimb44020036 35723322 PMC8928950

[B21] Kanehisa M. Furumichi M. Sato Y. Kawashima M. Ishiguro-Watanabe M. (2023). KEGG for taxonomy-based analysis of pathways and genomes. Nucleic Acids Res. 51 (D1), D587–d592. 10.1093/nar/gkac963 36300620 PMC9825424

[B22] Lee J. J. Cho W. K. Kwon H. Gu M. Ma J. Y. (2014). Galla rhois exerts its antiplatelet effect by suppressing ERK1/2 and PLCβ phosphorylation. Food Chem. Toxicol. 69, 94–101. 10.1016/j.fct.2014.03.032 24685825

[B23] Lee H. Y. Ghimeray A. K. Yim J. H. Chang M. S. (2015). Antioxidant, collagen synthesis activity in Vitro and clinical test on anti-wrinkle activity of formulated cream containing Veronica officinalis extract. J. Cosmet. Dermatological Sci. Appl. 05 (01), 45–51. 10.4236/jcdsa.2015.51006

[B24] Li Q. Hoppe T. (2023). Role of amino acid metabolism in mitochondrial homeostasis. Front. Cell Dev. Biol. 11, 11–2023. 10.3389/fcell.2023.1127618 PMC10008872 36923249

[B25] Liu W. Yan F. Xu Z. Chen Q. Ren J. Wang Q. (2022). Urolithin A protects human dermal fibroblasts from UVA-Induced photoaging through NRF2 activation and mitophagy. J. Photochem. Photobiol. B Biol. 232, 112462. 10.1016/j.jphotobiol.2022.112462 35567884

[B26] Ma Y. Yu L. Di P. Zhu X. Ma X. Kong W. (2026). Fungal-fungal interaction between Sanghuangporus vaninii and its endophytic Fusarium solani rewires host secondary metabolism to boost bioactive metabolite production. Microb. Cell Factories 25 (1), 135. 10.1186/s12934-026-02994-z PMC13214342 41935273

[B27] Martic I. Papaccio F. Bellei B. Cavinato M. (2023). Mitochondrial dynamics and metabolism across skin cells: implications for skin homeostasis and aging. Front. Physiol. 14, 1284410. 10.3389/fphys.2023.1284410 38046945 PMC10693346

[B28] Mazzei P. Piccolo A. (2012). 1H HRMAS-NMR metabolomic to assess quality and traceability of mozzarella cheese from Campania Buffalo milk. Food Chem. 132 (3), 1620–1627. 10.1016/j.foodchem.2011.11.142 29243658

[B29] Mero A. A. Ojala T. Hulmi J. J. Puurtinen R. Karila T. A. Seppälä T. (2010). Effects of alfa-hydroxy-isocaproic acid on body composition, DOMS and performance in athletes. J. Int. Soc. Sports Nutr. 7, 1. 10.1186/1550-2783-7-1 20051111 PMC2818616

[B30] Muñoz R. Rivas B. d.l. Rodríguez H. Esteban-Torres M. Reverón I. Santamaría L. (2024). Food phenolics and Lactiplantibacillus plantarum. Int. J. Food Microbiol. 412, 110555. 10.1016/j.ijfoodmicro.2023.110555 38199014

[B31] Naidoo K. Hanna R. Birch-Machin M. A. (2018). What is the role of mitochondrial dysfunction in skin photoaging? Exp. Dermatol 27 (2), 124–128. 10.1111/exd.13476 29197123

[B32] Niccolini B. Riente A. Hatem D. Bottoni P. Pizzoferrato M. Tringali G. (2025). Polydatin prevents UVA-induced damage in human dermal fibroblasts by maintaining mitochondrial integrity. Cells 14 (21), 1702. [Online]. 10.3390/cells14211702 41227347 PMC12607506

[B33] Pahalagedara A. S. N. W. Flint S. Palmer J. Brightwell G. Gupta T. B. (2022). Antibacterial efficacy and possible mechanism of action of 2-hydroxyisocaproic acid (HICA). PLOS ONE 17 (4), e0266406. 10.1371/journal.pone.0266406 35363830 PMC8975099

[B34] Pandel R. Poljšak B. Godic A. Dahmane R. (2013). Skin photoaging and the role of antioxidants in its prevention. ISRN Dermatol 2013, 930164. 10.1155/2013/930164 24159392 PMC3789494

[B35] Park J. M. Park K. J. (2010). The anti-wrinkle effects and whitening effects of Galla rhois. J. Korean Med. Ophthalmol. Otolaryngology Dermatology 23 (1), 135–148. Available online at: https://kci.go.kr/kciportal/landing/article.kci?arti_id=ART001438878#1.

[B36] Park P.-H. Hur J. Gogo G. Kim Y.-C. Jeong G.-S. Sohn D.-H. (2011). Inhibition of nitric oxide production by ethyl digallates isolated from Galla rhois in RAW 264.7 macrophages. Biomol. Ther. 19, 419–424. 10.4062/biomolther.2011.19.4.419

[B37] Parzonko A. Kiss A. K. (2019). Caffeic acid derivatives isolated from Galinsoga parviflora herb protected human dermal fibroblasts from UVA-Radiation. Phytomedicine 57, 215–222. 10.1016/j.phymed.2018.12.022 30785017

[B38] Quan T. Li R. Gao T. (2025). Role of mitochondrial dynamics in skin homeostasis: an update. Int. J. Mol. Sci. 26 (5), 1803. [Online]. 10.3390/ijms26051803 40076431 PMC11898645

[B39] Quintana-Cabrera R. Scorrano L. (2023). Determinants and outcomes of mitochondrial dynamics. Mol. Cell 83 (6), 857–876. 10.1016/j.molcel.2023.02.012 36889315

[B40] Rittié L. Fisher G. J. (2015). Natural and sun-induced aging of human skin. Cold Spring Harb. Perspect. Med. 5 (1), a015370. 10.1101/cshperspect.a015370 25561721 PMC4292080

[B41] Ryu D. Mouchiroud L. Andreux P. A. Katsyuba E. Moullan N. Nicolet-dit-Félix A. A. (2016). Urolithin A induces mitophagy and prolongs lifespan in *C. elegans* and increases muscle function in rodents. Nat. Med. 22 (8), 879–888. 10.1038/nm.4132 27400265

[B42] Shirzad M. Javad H. Elahe M. Modarressi M. H. (2018). Anti-elastase and anti-collagenase potential of lactobacilli exopolysaccharides on human fibroblast. Artif. Cells, Nanomedicine, Biotechnol. 46(Suppl. 1)**,** 1051–1061. 10.1080/21691401.2018.1443274 29486611

[B43] Sivanand S. Vander Heiden M. G. (2020). Emerging roles for branched-chain amino acid metabolism in cancer. Cancer Cell 37 (2), 147–156. 10.1016/j.ccell.2019.12.011 32049045 PMC7082774

[B44] Sreedhar A. Aguilera-Aguirre L. Singh K. K. (2020). Mitochondria in skin health, aging, and disease. Cell Death and Dis. 11 (6), 444. 10.1038/s41419-020-2649-z 32518230 PMC7283348

[B45] Stout R. Birch-Machin M. (2019). Mitochondria's role in skin ageing. Biol. (Basel) 8 (2), 29. 10.3390/biology8020029 31083540 PMC6627661

[B46] Tian F. Li B. Ji B. Yang J. Zhang G. Chen Y. (2009). Antioxidant and antimicrobial activities of consecutive extracts from galla chinensis:the polarity affects the bioactivities. Food Chem. 113 (1), 173–179. 10.1016/j.foodchem.2008.07.062

[B47] Tseng C. Y. Salguero J. A. Breidenbach J. D. Solomon E. Sanders C. K. Harvey T. (2025). Evaluation of normalization strategies for mass spectrometry-based multi-omics datasets. Metabolomics 21 (4), 98. 10.1007/s11306-025-02297-1 40593232 PMC12214035

[B48] Wang R. Novick S. J. Mangum J. B. Queen K. Ferrick D. A. Rogers G. W. (2015). The acute extracellular flux (XF) assay to assess compound effects on mitochondrial function. SLAS Discov. 20 (3), 422–429. 10.1177/1087057114557621 25381255

[B49] Wang Y. Mi Z. Li C. Pan L. Sun Z. Sun T. (2019). Metabolites profile analysis of fermented milk with Lactobacillus plantarum P-8 based on ultra-performance liquid chromatography-quadrupole-time of flight mass spectrometry (UPLC-Q-TOF-MS). Sci. Technol. Food Industry 40 (11), 152–160. 10.13386/j.issn1002-0306.2019.11.026

[B50] Wang Z. Liu X. Liu S. Zhou W. Chen T. Gao S. (2025). Function identification of a lactonase gene lac1563 involved in producing urolithin A of Limosilactobacillus fermentum FUA033. J. Agric. Food Chem. 73 (11), 6795–6806. 10.1021/acs.jafc.4c10908 40062842

[B51] Wang J. Gu J. Li X. Sun H. Yang X. (2026). The central role of the AMPK/SIRT1/PGC-1α signaling axis in skeletal muscle physiology and pathology and its targeted therapeutic strategies. Pharmaceuticals 19 (7), 1056. 10.3390/ph19071056 PMC13414853 42515737

[B52] Wu D. Jiang S. Li C. Wang X. Ma Y. Wang P. (2026). Mitochondrial dynamics in cellular senescence: mechanisms, context dependence, and therapeutic potential. Aging Disease. 10.14336/ad.2026.0323 42295095

[B53] Ye R. Wang Q. Du L. Li L. Hu F. (2026). Cornus officinalis fruit extract as an AMPK-associated mitochondrial bioenergetic modulator in skin aging models. Biomedicines 14 (2), 403. [Online]. 10.3390/biomedicines14020403 41751302 PMC12938615

[B54] Yuan X. Li H. Lee J. S. Lee D. H. (2025). Role of mitochondrial dysfunction in UV-Induced photoaging and skin cancers. Exp. Dermatol. 34 (5), e70114. 10.1111/exd.70114 40318065

[B55] Zhang C. Gao X. Li M. Yu X. Huang F. Wang Y. (2023a). The role of mitochondrial quality surveillance in skin aging: focus on mitochondrial dynamics, biogenesis and mitophagy. Ageing Res. Rev. 87, 101917. 10.1016/j.arr.2023.101917 36972842

[B56] Zhang M. Cui S. Mao B. Zhang Q. Zhao J. Zhang H. (2023b). Ellagic acid and intestinal microflora metabolite urolithin A: a review on its sources, metabolic distribution, health benefits, and biotransformation. Crit. Rev. Food Sci. Nutr. 63 (24), 6900–6922. 10.1080/10408398.2022.2036693 35142569

[B57] Zheng J. Wittouck S. Salvetti E. Franz C. Harris H. Mattarelli P. (2020). A taxonomic note on the genus lactobacillus: description of 23 novel genera, emended description of the genus lactobacillus beijerinck 1901, and union of lactobacillaceae and leuconostocaceae. Int. J. Syst. Evol. Microbiol. 70, 2782–2858. 10.1099/ijsem.0.004107 32293557

[B58] Zheng Q. Jin X. Nguyen T. T. M. Yi E. J. Park S. J. Yi G. S. (2025). Autophagy-enhancing properties of Hedyotis diffusa extracts in HaCaT keratinocytes: potential as an anti-photoaging cosmetic ingredient. Molecules 30 (2), 261. 10.3390/molecules30020261 39860131 PMC11767327

[B59] Zhou X. Wang N. Wei J. Li G. Lue Z. Liu C. (2025). Pterostilbene mitigates the senescence of human dermal fibroblast cells by enhancing mitochondrial quality. Front. Pharmacol. 16, 16–2025. 10.3389/fphar.2025.1732154 41438513 PMC12719513

