# Supplementary material for: Enhanced mitochondrial respiratory metabolism in UV-damaged HDF cells based on metabolomics analysis of fermented Galla rhois gallnut extract
Source: Front Pharmacol. 2026 Oct 27;17:1922913. doi: 10.3389/fphar.2026.1922913 (PMC13749433; doi:10.3389/fphar.2026.1922913)
Supplement: Supplementary file 1 [file Supplementaryfile1.docx]

Figure S1 The effect of different concentration of fermented *Galla rhois* gallnut extract on cellular mitochondrial function (n=3). (A) Cellular OCR curve; (B) Basal respiration; (C) ATP production; (D) Maximal respiration; (E) Spare respiration capacity. Letters (a-c) indicate significant differences among groups (*p* < 0.05): groups not sharing a letter differ significantly; groups sharing a letter are not significantly different.

Table S2 Compounds recognition from differential metabolites between FGRE and GRE (n=10).

| Metabolite | Mode | Formula | Adducts | M/Z | Authentic Standard | RT_sample_ (min) | ΔRT (min) | Δppm | MS/MS Spectral Matching Score | Overall Identification Score | Level | FGRE vs GRE |
| --- | --- | --- | --- | --- | --- | --- | --- | --- | --- | --- | --- | --- |
| L-Aspartic acid | Negative | C_4_H_7_NO_4_ | [M-H]^-^ | 132.0303 | Yes | 0.5070 | -0.06 | 0.62 | 99.4 | 59.3 | 1 | Down |
| L-Glutamic acid | Negative | C_5_H_9_NO_4_ | [M-H]^-^ | 146.0460 | Yes | 0.5310 | -0.09 | -3.25 | 99.4 | 59.2 | 1 | Down |
| Leucinic Acid | Negative | C_6_H_12_O_3_ | [2M-H]^-^ | 263.0522 | Yes | 4.7372 | 0.19 | 0.47 | 40.8 | 46.6 | 2 | Up |
| Afzelechin | Negative | C_15_H_14_O_5_ | [M-H]^-^ | 273.0774 | Yes | 5.6564 | 1.43 | 2.14 | 42.0 | 44.7 | 2 | Down |
| Ser-Ile | Positive | C_9_H_18_N_2_O_4_ | [M+H]^+^ | 219.1333 | Yes | 1.8098 | / | -2.75 | 57.3 | 49.9 | 2 | Down |
| Ile-Asn-Val | Positive | C_15_H_28_N_4_O_5_ | [M+H]^+^ | 345.2125 | Yes | 3.3285 | / | -2.31 | 38.9 | 45.4 | 2 | Down |
| His-Gly-Glu | Positive | C_13_H_19_N_5_O_6_ | [M+H]^+^ | 342.1385 | Yes | 0.5390 | / | -6.77 | 71.4 | 51.5 | 2 | Down |
| Cyclokievitone | Negative | C_20_H_18_O_6_ | [M-H]^-^ | 353.1009 | Yes | 4.9326 | / | -6.21 | 56.4 | 48.7 | 2 | Up |
| Choline glycerophosphate | Positive | C_8_H_20_NO_6_P | [M+H]^+^ | 258.1092 | Yes | 0.5160 | / | -3.47 | 70.1 | 52.4 | 2 | Down |
| Arg-Val-Phe | Positive | C_20_H_32_N_6_O_4_ | [M+H]^+^ | 421.2582 | Yes | 4.4801 | / | 5.85 | 51.1 | 46.2 | 2 | Up |
| Ala-Leu | Negative | C_9_H_18_N_2_O_3_ | [M-H]^-^ | 201.1246 | Yes | 2.6624 | / | 0.45 | 59.3 | 50.9 | 2 | Down |
| Ala-Ile | Positive | C_9_H_18_N_2_O_3_ | [M+H]^+^ | 203.1384 | Yes | 1.9744 | / | -3.07 | 69.1 | 52.4 | 2 | Down |
| Leu-Ser-Ile | Positive | C_15_H_29_N_3_O_5_ | [M+H]^+^ | 332.2171 | Yes | 4.2314 | / | -2.60 | 35.3 | 44.0 | 2 | Down |
| Leu-Asp-Val | Positive | C_15_H_27_N_3_O_6_ | [M+H]^+^ | 346.1966 | Yes | 3.4010 | / | -2.03 | 35.9 | 43.7 | 2 | Down |
| Vignatic acid A | Negative | C_30_H_39_N_3_O_7_ | [M+Hac-H]^-^ | 612.2895 | No | 5.7229 | / | -5.79 | 63.4 | 48.8 | 3 | Up |
| Val-Asn | Positive | C_9_H_17_N_3_O_4_ | [M+Na-H_2_O]^+^ | 236.1000 | No | 0.6745 | / | -4.74 | 42.3 | 45.1 | 3 | Up |
| Urolithin M5 | Negative | C_13_H_8_O_7_ | [M-H]^-^ | 275.0178 | No | 2.1892 | / | -6.54 | 44.1 | 46.2 | 3 | Up |
| Urolithin A | Negative | C_13_H_8_O_4_ | [M-H]^-^ | 227.0335 | No | 3.0304 | / | -4.04 | 43.6 | 45.7 | 3 | Up |
| Thr-Ile | Positive | C_10_H_20_N_2_O_4_ | [M+H]^+^ | 233.1489 | No | 2.2217 | / | -2.78 | 54.1 | 49.1 | 3 | Down |
| Riboflavin reduced | Negative | C_15_H_16_N_4_O_6_ | [M-H]^-^ | 347.1026 | No | 4.7340 | / | 8.44 | 55.7 | 45.8 | 3 | Up |
| L-Arogenate | Negative | C_10_H_12_NO_5_ | [M+FA-H]^-^ | 271.0706 | No | 3.3362 | / | 3.97 | 49.2 | 45.3 | 3 | Down |
| Chakanoside I | Negative | C_14_H_18_O_7_ | [M-H_2_O-H]^-^ | 279.0898 | No | 0.5602 | / | 8.15 | 48.4 | 44.9 | 3 | Up |
| 6-Geranylnaringenin | Negative | C_25_H_28_O_5_ | [M+Na-2H]^-^ | 429.1648 | No | 4.7422 | / | -8.77 | 48.1 | 43.8 | 3 | Up |
| 2-Pentanone | Negative | C_5_H_10_O | [M-H]^-^ | 85.0660 | No | 4.7308 | / | 0.88 | 40.6 | 47.6 | 3 | Up |

The identification criteria were as follow: (i) mass accuracy within 10 ppm (Mass error); (ii) MS/MS spectral matching score ≥ 35 out of 100 (Fragmentation score); (iii) overall identification score ≥ 36 out of 60, which integrates mass accuracy (20 points), MS/MS fragment matching (20 points), and isotopic distribution matching (20 points). Level 1 compounds were verified using authentic standards with |ΔRT| ≤ 0.1 min (ΔRT=RT_sample_-RT_standard_) and met all the thresholds described above. Level 2 (putative) compounds likewise met the thresholds above and were matched against authentic standards, but did not satisfy the full Level 1 retention-time criterion, i.e., the retention-time deviation exceeded the 0.1-min tolerance (|ΔRT| > 0.1 min) or no ΔRT value was available for comparison. Level3 compounds with MS/MS spectral matching scores ≥ 40 but without authentic standards were considered putative.

Figure S3 Molecular docking results of leucinic acid with LDH: (A) Comparison of the active pockets between hLDHD (blue) and mLDHD (yellow); (B) Docking conformation of hLDHD (gray) with D-leucinic acid (pink); (C) Docking conformation of hLDHA (gray) with L-leucinic acid (pink). (D) Molecular docking and molecular dynamics simulation results. The yellow sticks represent the cofactor.

**Method:** The structure model of human D-lactate dehydrogenase (hLDHD) was predicted using AlphaFold3, while the structure model of human L-lactate dehydrogenase A (hLDHA) was derived from crystal structure (PDB ID: 4JNK). Preprocessing of both protein structures involved structure optimization, hydrogen atom addition, and charge correction. D-leucic acid and L-leucic acid, as the docking ligands, was preprocessed using AutoDock Tools. The docking site for hLDHD was defined based on the position of the D-leucic acid in the crystal structure of mouse D-lactate dehydrogenase (mLDHD) (PDB ID: 8JDQ). The center coordinates of the docking site were (-2.55, 9.18, 0.21), with a docking radius of 10 Å. For hLDHA, the docking site was defined according to the binding position of the pyruvate in the crystal structure. The center coordinates of the docking site were (52.12, -10.63, 39.63), with a docking radius of 10 Å. Molecular docking procedures were performed using the Autodock Vina.

The optimal docking conformation was subjected to molecular dynamics simulation using the Gromacs 2019 on the MaxFlow platform. The AMBER14SB force field was selected for standard residues, while the GAFF2 force field was applied for non-standard residues. The TIP3P model was employed as the water molecule. Solvation was performed to simulate an aqueous environment using explicit water molecules. Sodium (Na⁺) and chloride (Cl⁻) ions were added to neutralize the system charge. The geometry and energy of the system were optimized using the steepest descent algorithm to reach a local energy minimum. Temperature equilibration was subsequently performed in the NVT ensemble at 298 K for 0.5 ns. Finally, a 50 ns molecular dynamic simulation was performed using the NPT ensemble, with the temperature coupling method set to the Berendsen thermostat and the pressure coupling method to the Berendsen barostat. MD simulation analysis was also performed using the MaxFlow platform. Structural analysis and figure preparation were carried out using chimera X.

Figure S4 The effect of different concentration of UroM5 and UroA on cellular mitochondrial function (n=3). (A) Cell viability; (B) Cellular OCR curve; (C) Basal respiration; (D) ATP production; (E) Maximal respiration; (F) Spare respiration capacity. Letters (a-d) indicate significant differences among groups (*p* < 0.05): groups not sharing a letter differ significantly; groups sharing a letter are not significantly different.

**Methods:** Different concentration of UroA and UroM5 were explored the effect on mitochondrial respiratory by the methods as part 2.4 (Mitochondrial respiratory measurement in UVB-induced HDFs). All experiments were performed with at least three biological replicates per experimental group. For each biological replicate, more than three technical replicate wells were prepared per condition, and the measurements from these technical wells were averaged to yield a single data point per biological replicate prior to statistical analysis.

Figure S5 The effect of different concentration of UroA (A) and UroM5 (B) on ROS inhibition in UVB-induced HaCaT cells (n=3). Letters (a-d) indicate significant differences among groups (*p* < 0.05): groups not sharing a letter differ significantly; groups sharing a letter are not significantly different.

**Method:** The HaCaT cells were randomly divided into groups and cultured in DMEM medium supplemented with 10% fetal bovine serum at 5% CO2 and 37℃ for 24h. And the sample groups were added different concentration UroA and UroM5 intervention while other groups with no intervention for 24h. Then, the cells in UVB group and sample groups were irradiated with UVB in PBS with a dose of 180 mJ/cm^2^. UVB (NB-UVB) used are generated by a Narrow-Band UVB lamp (Philips, TL20W/01 lamps) with a peak at 311 nm. The emitted radiation was checked under the microplate using a radiometer (Solar Light PMA2100, USA). The cells were washed 2~3 times with PBS, and added 500 μL of 10 μM DCFH-DA probe to each well. Then, the cells were incubated the cells at 37 ℃ and 5% CO₂ for 30 min and removed the probe-containing medium. And the cells were detached by adding 500 μL of 0.25% trypsin solution per well, collected by centrifugation, and resuspended in PBS. Fluorescence intensity was measured using a microplate reader at excitation/emission wavelengths of 488 nm and 525 nm, respectively, with real-time monitoring of the relative fluorescence intensity before and after stimulation. All experiments were performed with at least three biological replicates per experimental group.
